# Machine learning prediction of novel pectinolytic enzymes in *Aspergillus niger* through integrating heterogeneous (post-) genomics data

**DOI:** 10.1099/mgen.0.000674

**Published:** 2021-12-07

**Authors:** Mao Peng, Ronald P. de Vries

**Affiliations:** ^1^​ Fungal Physiology, Westerdijk Fungal Biodiversity Institute, & Fungal Molecular Physiology, Utrecht University, Utrecht, The Netherlands

**Keywords:** *Aspergillus niger*, machine learning, (post-)genomics, pectinolytic enzymes

## Abstract

Pectinolytic enzymes are a variety of enzymes involved in breaking down pectin, a complex and abundant plant cell-wall polysaccharide. In nature, pectinolytic enzymes play an essential role in allowing bacteria and fungi to depolymerize and utilize pectin. In addition, pectinases have been widely applied in various industries, such as the food, wine, textile, paper and pulp industries. Due to their important biological function and increasing industrial potential, discovery of novel pectinolytic enzymes has received global interest. However, traditional enzyme characterization relies heavily on biochemical experiments, which are time consuming, laborious and expensive. To accelerate identification of novel pectinolytic enzymes, an automatic approach is needed. We developed a machine learning (ML) approach for predicting pectinases in the industrial workhorse fungus, *Aspergillus niger*. The prediction integrated a diverse range of features, including evolutionary profile, gene expression, transcriptional regulation and biochemical characteristics. Results on both the training and the independent testing dataset showed that our method achieved over 90 % accuracy, and recalled over 60 % of pectinolytic genes. Application of the ML model on the *A. niger* genome led to the identification of 83 pectinases, covering both previously described pectinases and novel pectinases that do not belong to any known pectinolytic enzyme family. Our study demonstrated the tremendous potential of ML in discovery of new industrial enzymes through integrating heterogeneous (post-) genomimcs data.

## Data Summary

1. Genome and transcriptome data analysed in this work are available in the Mycocosm and GEO database, respectively. Individual accession numbers are listed in Table S1, available in the online version of the article.
2. Novel pectinolytic enzymes predicted in this study are listed in Table S6.
3. The curated sequence and expression features and source code for predicting pectinolytic enzymes have been deposited in GitHub (https://github.com/maopeng2018/pectinase_prediction).
4. Supplementary Material can be found in Figshare: https://doi.org/10.6084/m9.figshare.14958117.v1 [1].

**Impact Statement**

Pectinolytic enzymes are involved in breaking down pectin, a complex and abundant plant cell-wall polysaccharide. Due to their important biological function and increasing industrial applications, discovery of novel pectinolytic enzymes has received global interest. However, traditional enzyme discovery and characterization is time consuming and expensive. This paper describes a machine learning (ML) approach for predicting pectinases in the industrial workhorse fungus, *Aspergillus niger*. The prediction model integrated diverse features ranging from evolutionary profile, gene expression, transcriptional regulation to biochemical characteristics, which enabled it to successfully identify both previously described pectinases and novel pectinase candidates. The prediction results provide valuable resources for further experimental validation and exploration of the important function of novel enzyme candidates. In addition, our study provides a framework for discovery of other novel microbial industrial enzymes through ML analysis of continually increasing heterogeneous (post-)genomics data.

## Introduction

Pectin is a heteropolymer that is abundantly present in the plant cell wall [2]. It is composed of four structural elements: homogalacturonan (HGA), xylogalacturonan (XGA), rhamnogalacturonan I (RG-I) and rhamnogalacturonan II (RG-II). In nature, many bacteria and fungi can efficiently degrade pectin by secreting a variety of pectinolytic enzymes. These enzymes play an essential role in facilitating microbial organisms to release metabolizable sugars from recalcitrant plant biomass and helping microbial pathogens to penetrate into the plant host [2, 3]. Pectinases mainly belong to three carbohydrate active enzyme (CAZy) groups [4] on the basis of their mode of action: glycoside hydrolases (GHs, EC 3.2.1.-), polysaccharide lyases (PLs, EC 4.2.2.-) and carbohydrate esterases (CEs, EC 3.1.1.-). In more detail, the fungal pectin backbone cleaving enzymes consist of polygalacturonases and rhamnogalacturonases (GH28), pectin and pectate lyases (PL1, PL3, PL9), and rhamnogalacturonan lyases (PL4, PL26). The fungal enzymes that act on the RG-I side chains include endo- and exoarabinanases (GH43, GH93), α-arabinofuranosidases (GH3, GH43, GH51, GH54, GH62), galactanases (GH5, GH30, GH53), and β-galactosidases (GH1, GH2, GH35, GH43). In addition, several fungal esterases have an accessory role in pectin degradation, e.g. pectin methylesterases (CE8), pectin acetylesterases (CE12 and CE13), rhamnogalacturonan acetylesterases (CE12) and feruloyl esterases (CE1).

Microbial pectinases have been widely used in various industries such as processing of wine, food, animal feed, and production of paper, pulp and biofuel [5, 6]. The filamentous fungus *Aspergillus niger* is an industrial workhorse that has been widely exploited as a cell factory to produce organic acids and various enzymes, including pectinases [7].

The discovery of novel pectinolytic enzymes and expanding their potential industrial applications have attracted broad research interest. However, traditional approaches for identifying novel fungal enzymes mainly rely on homology searches using known enzymes, transcriptomics analysis of fungi grown under a limited set of conditions and/or extensive biochemical experiments which are laborious and expensive. So far, only 37 *A*. *niger* pectinolytic genes have been experimentally characterized. To accelerate novel enzyme identification, an automatic approach is needed.

Recent (post-)genomics studies in fungal pectinolytic enzymes have greatly enhanced our understanding of the evolution, transcriptional expression and regulation of fungal pectinolytic genes. For instance, one study showed that differences in the growth of various fungi on pectin were partly caused by the different pectinolytic enzymes encoded in their genome [8]. Increasing applications of transcriptomics studies have revealed the specific expression patterns of pectinolytic genes during growth on both simple sugars and crude plant biomass [9, 10]. In addition, several important transcriptional regulators involved in regulating pectinolytic genes have been discovered, including GaaR [11], GaaX [12], AraR [13] and RhaR [14]. These continuously increasing omics data together with emerging bioinformatics methods have shown tremendous potential for the discovery of novel enzymes [15–17].

In this study, we developed a machine learning (ML) approach for predicting pectinases in *A. niger* by jointly considering genomic, transcriptomic, regulation and biochemical features. The ML model showed good performance in both training and independent testing datasets, as well as in identifying novel enzyme candidates. Our study demonstrated that the integrative analysis of heterogeneous (post-)genomics features using the ML method is a promising approach for finding novel industrially important enzymes.

## Methods

**Fig. 1.**
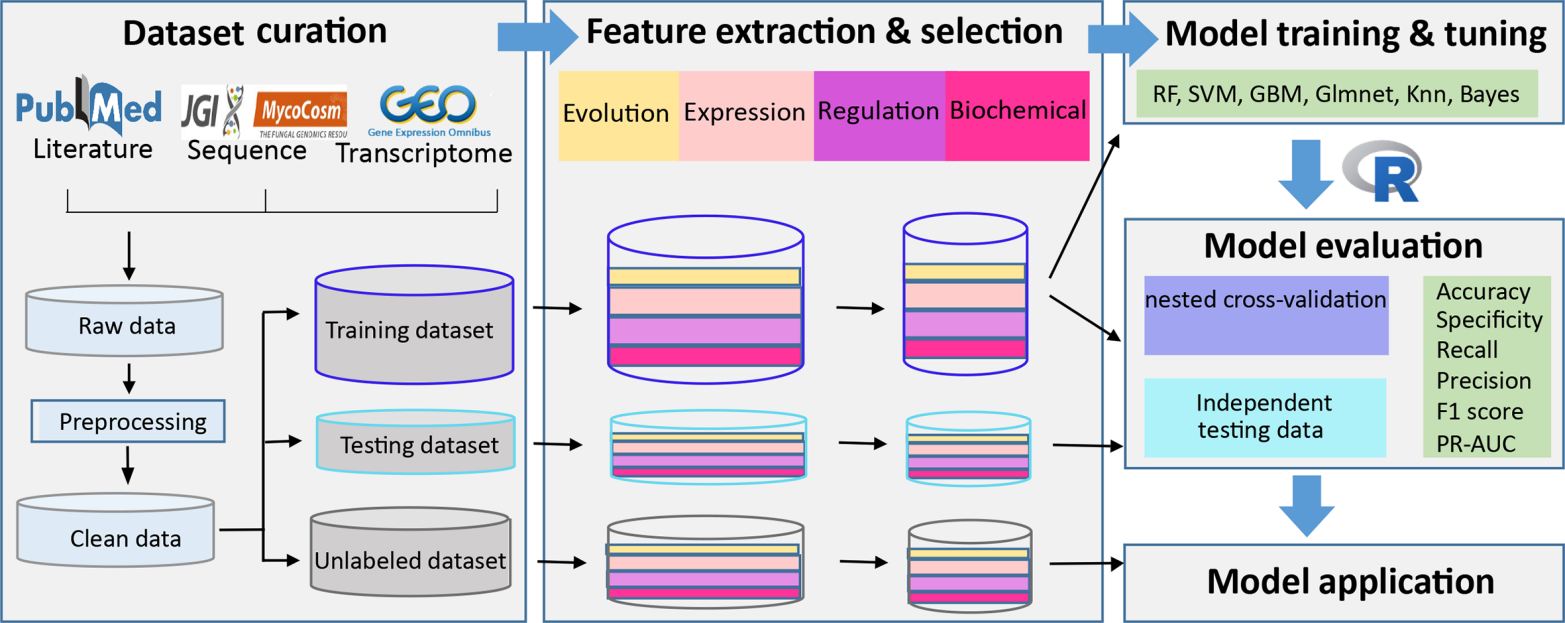
Flowchart of the machine learning (ML)-based approach. The workflow is composed of five steps. First, we performed extensive literature searches to collect experimentally characterized pectinolytic and non-pectinolytic genes that were used as positive and negative references. Related sequence and expression profiles were obtained from Mycocosm and the GEO database. Subsequently, four types of features derived from the evolutionary profile, gene expression, gene regulation and biochemical characteristics of encoding proteins were extracted. Then the feature encodings were further filtered and selected as input for training the classifiers, resulting in their corresponding prediction models. Finally, we selected the model with the best performance based on rigorous evaluation, and applied it for genome-wide prediction of pectinolytic genes.

The workflow for the prediction of pectinolytic genes is depicted in Fig. 1. The analysis process includes five major steps, which are described in the following subsections.

### Data collection and preprocessing

The construction of a high-quality benchmark dataset for training and validating the model is a prerequisite to perform a successful ML prediction. In this study, we performed extensive literature searches and collected 37 experimentally characterized extracellular pectinolytic enzymes as positive training instances. The negative training instances were constructed with 310 non-pectinolytic genes encoding five different types of secreted proteins, but with a predicted or characterized function that is not involved in pectin degradation. More specifically, the negative gene set includes 34 experimentally characterized genes encoding enzymes for degrading non-pectin polysaccharides (e.g. starch, inulin, cellulose and hemicellulose), 53 genes encoding peptidases involved in protein lysis, 81 genes encoding transporters, 88 CAZy genes annotated with non-plant biomass degradation and 59 cytochrome P450 genes. The sequences and functional annotation related to secretion signal peptide (filtered with SignalP [18] hmm-score >0.8), Pfam domains, transporters, and the CAZy and protease were obtained from Mycocosm [19]. To avoid possible false negative prediction by using SignalP prediction, the final secretome also combined the secretion proteins predicted from the online Phobius tool [20] (https://phobius.sbc.su.se/). We used the full set of proteome sequences of *A. niger* as input and set the default parameters during the Phobius prediction. The polysaccharide-degrading specificity of CAZy was derived from the literature [21]. We randomly split negative genes into training and independent testing set in a ratio 3 : 1. The positive genes used in the independent test set came from a previous study based on sequence homology searches and transcriptome analysis [9], which described 60 pectinolytic genes including both the predicted and the characterized genes. After removing 37 genes that were already used in the training set and 14 pseudo genes without any detectable expression in the transcriptome data, only nine genes were included in our testing set. As a result, the training set includes 37 pectinolytic genes and 233 non-pectinolytic genes, and the independent testing set includes nine previously predicted pectinolytic genes and 77 non-pectinolytic genes.

### Feature extraction and selection

We extracted four different categories of features: evolutionary profile, gene expression, gene regulation and biochemical-based features (Table S1). In total, 68 initial features were obtained (Table S2). After feature extraction, we used feature selection to remove redundant and unimportant variables to improve the performance of the ML classification.

### Features derived from evolutionary information

We calculated two types of evolutionary features, the conservation score and co-occurrence score. The conservation score was defined by the ratio of the orthologue of each *A. niger* gene present in 10 selected fungi species (including *A. niger*), with a minimum value of 0.1 and maximum value of 1 to indicate the orthologue gene is only present in *A. niger* or present in all 10 species, respectively. Orthologues were identified by OrthoMCL [22] with parameters: E-value 1E−5, inflation level 1.5 and sequence coverage 60 %. The 10 selected fungus species were derived from a previous study [23]. The co-occurrence score denotes the similarity of the presence and absence profile of orthologous genes in selected genomes, which is commonly used to infer functional links between genes [24]. For each *A. niger* gene against a known pectinolytic gene (excluding two identical genes), we calculated a Jaccard index of their presence and absence profile of orthologous genes in the selected genomes, indicating the similarity in phylogenetic profile of two compared genes. This resulted in 37 and 36 Jaccard scores for each non-characterized pectinolytic gene and each known pectinolytic gene, respectively. The median, maximum and standard deviation of those Jaccard scores for each gene were used for ML analysis.

### Features derived from transcriptome data

We obtained gene expression profiles of *A. niger* grown on a broad range of monosaccharides, polysaccharides and crude plant biomass (including pectin) from two previous transcriptomics studies [10, 25]. The minimum, maximum, mean and variation for the expression values of each gene across all the conditions were calculated. In addition, because many pectinolytic genes were observed to cluster together in their transcriptome profile [10], the co-expression profile as a common feature used in gene function prediction was analysed here [15, 26, 27]. We calculated co-expression scores between each *A. niger* gene with the 37 well-characterized pectinolytic genes. Two common co-expression scores were calculated: the Pearson correlation coefficient (PCC) and the mutual information (MI) [28]. This resulted in 37 and 36 PCC and MI scores for each non-characterized pectinolytic gene and each known pectinolytic gene, respectively. The median and maximum PCC scores and the median and minimum MI scores for each gene were used for ML prediction.

### Features derived from gene regulation profile

The expression of fungal extracellular enzymes is precisely regulated by a series of transcription factors (TFs) [29]. These regulators include XlnR [30], AraR [31], RhaR (Kowalczyk *et al*., 2017), GaaR (Alazi *et al*., 2016) and AmyR [32], controlling production of enzymes involved in degrading cellulose, hemicellulose, pectin and starch, respectively. CreA functions as a general repressor inhibiting production of lignocellulolytic enzymes when a carbon source is easily available [33]. Therefore, integration of the regulation pattern of those regulators can help distinguish the pectinolytic genes and non-pectinolytic genes. Here, we compared transcriptomic data of several TF mutants and their corresponding reference strains obtained from several previous studies [10–13, 33]. Values of 1, –1 and 0 were used to indicate genes that were up- or down-regulated, or have no significant changes when comparing the TF mutant and the reference strain. In addition, the frequencies of binding sites of those TFs (if known) in the promoters of the selected genes (1000 bp upstream region) were extracted.

### Features derived from biochemical properties of encoding proteins

We extracted three common biochemical-based features, protein length, isoelectric point (pI) and number of Pfam domains for the proteins encoded by each gene. Protein sequences and Pfam domains were obtained from Mycocosm [19], and pI was extracted from the Proteome-pI database [34].

### Feature selection and importance ranking

Feature selection is crucial to lower the complexity of the ML model and improve its performance [35]. We first removed redundant features based on PCC. For any two features with a PCC >0.7, we only kept one of them for further analysis (Fig. S1, Table S1). Subsequently, the Recursive Feature Elimination method provided by the R package ‘mlr3fselect’ in the ‘mlr3’ framework was used to select features [36] based on the re-balanced data obtained from the down-sampling method, using the Random Forest algorithm [37] and evaluated by nested cross-validation (CV) . Variable importance measures for the final selected Random Forest model was determined with the ‘impurity’ method implemented in the Ranger package [38]. Details of the exclusion and inclusion of features during each feature selection step are shown in Table S1.

### Model training and optimization

To select the best model, we have compared the prediction results from six classical ML classifiers: Random Forest (RF), Support Vector Machine (SVM), Gradient Boosting Machines (GBM), Lasso and Elastic-Net Regularized Generalized Linear Models (GLMnet), k-Nearest Neighbours (kNN) and Naive Bayes (NB). All these models are well implemented in the ‘mlr3’ R package [36], which provides a unified interface for creating various ML models. The nested CV was performed to choose the hyperparameters and estimate the performance of the resulting model using the R package ‘mlr3tuning’ in the ‘mlr3’ framework [36]. The inner CV was used to tune the hyper-parameters, and the outer CV was used to assess the performance of the classifier with each optimal parameter setting from the inner CV. We report the average and standard deviation of performance measures in out-CV as an estimate of the generalization performance. The final model was built by using the optimal hyperparameter on the whole training dataset. The search space of the grid search and the optimized hyperparameter setting based on nested CV results are listed in Table S3.

Because there are more non-pectinase genes than pectinase genes in the training set, we constructed a balanced dataset by three different balancing methods: down-sampling, over-sampling and the SMOTE algorithm [39] implemented in R package ‘mlr3’ [36]. The simple down-sampling method performed best and was used in the final model training and tuning.

### Evaluation of model performance

#### Measurement methods

The performance of the models was rigorously assessed by two standard methods: nested CV and an independent test. The nested CV procedure provides a unbiased estimation of model performance compared to classical CV [40]. Given the relatively small size of the training set, we performed nested CV with five inner and three outer iterations. The independent test was conducted using the independent dataset. Since the independent dataset has no overlap with the training dataset, it represents a more rigorous validation of the prediction models.

#### Evaluation metrics

A set of five measures that are commonly used in bioinformatics studies were employed for quantifying the performance: accuracy (ACC), specificity (SP), Recall, Precision and F1 score. The relevant formulas for these measurements are as below:

$$Accuracy=\frac{\left(TP+TN\right)}{\left(TP+TN+FP+FN\right)}$$

$$Specificity=\frac{TN}{\left(TN+FP\right)}$$

$$Recall=\frac{TP}{\left(TP+FN\right)}$$

$$Precision=\frac{TP}{\left(TP+FP\right)}$$

$$F1score=\frac{2*(Precision*Recall)}{\left(Precision+Recall\right)}$$

where TP, TN, FP and FN represent the numbers of the true positive, true negative, false positive and false negative, respectively.

Morever, the area under precision-recall curves (PR-AUC) was calculated to provide an intuitive comparison of the performance of different models [41].

## Results

### Performance evaluation on the nested CV test

**Fig. 2.**
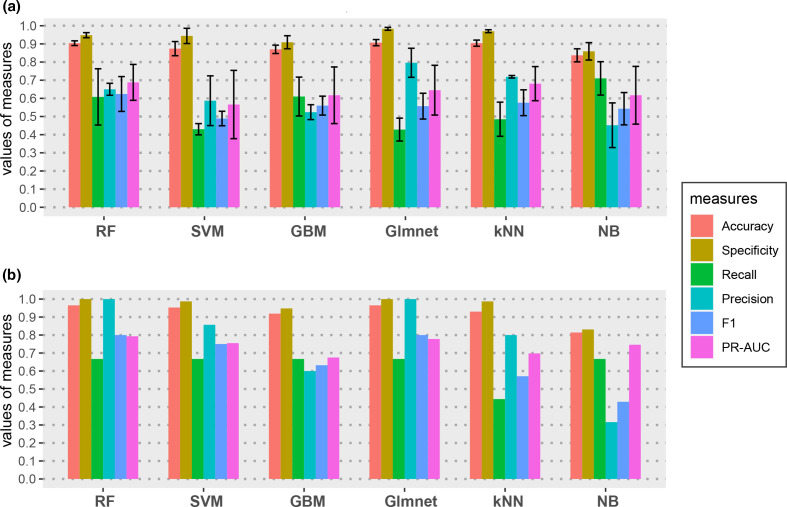
Comparison of the performance of different classifiers. (**a**) Performance of models on nested cross-validation (CV). The average and standard deviation of performance measures in out-CV are reported. (**b**) Performance of models with the optimized hyperparameter on the independent test set. Six classifiers were compared including Random Forest (RF), Support Vector Machine (SVM), Gradient Boosting Machines (GBM), Lasso and Elastic-Net Regularized Generalized Linear Models (GLMnet), k-Nearest Neighbours (kNN) and Naïve Bayes (NB).

As shown in Fig. 2 and Table S4, RF achieved a good performance based on nested CV. The SP and ACC of the RF model are above 0.9, and the precision, recall, F1 and PR-AUC are all above 0.6. The Glmnet and kNN classifiers performed less well, with ACC and SP >0.9, and a slightly higher precision, but with lower recall and PR-AUC compared to the RF model. The SVM, GBM and NB performed worse than the other three models on most of the evaluation metrics.

### Performance evaluation on an independent test

To further evaluate the proposed prediction models, we built the final classifiers on the full training set using the corresponding optimized hyperparameter settings from nested CV, and applied them to an independent dataset containing 77 non-pectiolytic genes and nine previously predicted pectinases. The RF and Glmnet model showed the best performance on most of evaluation metrics (Fig. 2 and Table S4). Both models correctly identified all negative instances and recalled 66.7 % of previous predicted pectinolytic genes. Based on the better performance of RF on both CV and the independent test than most of other ML models, as well as the superior recall rate of RF to Glmnet on the nested CV test, we chose it as the final model and applied it for genome-wide prediction of pectinolytic genes.

### Effect of feature selection

In total, 68 initial features were extracted (Table S2). After removing redundant features with high correlation, 45 features were kept for ML analysis. To lower the complexity of the ML model and improve its performance, we further applied the RF algorithm for feature selection [37], which selected 22 features used in the final ML models (Table S1). For most tested models the well-selected 22 features achieved a very close or even better performance than the 45 original features based on the nested CV test, and achieved even better performances on the independent test (Table S5). Since the feature selection reduced the training time and enhanced model generalization, all our final models were trained with the 22 selected features.

### Feature importance ranking

**Fig. 3.**
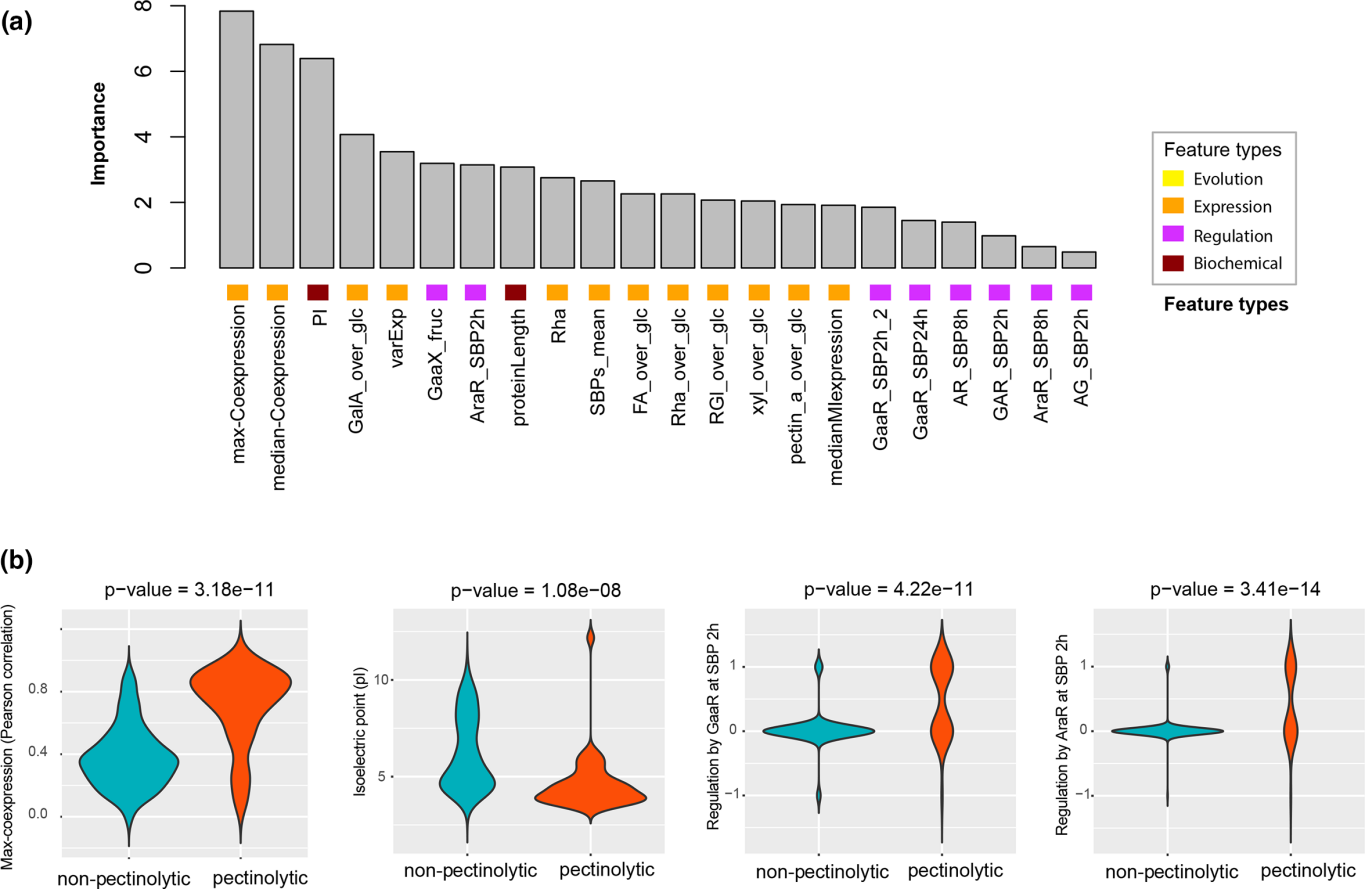
The importance of features for pectinolytic and non-pectinolytic gene prediction. (**a**) Importance of 22 features obtained from four different types of data for distinguishing pectinolytic and non-pectinolytic genes in the Random Forest model. (**b**) Distribution of four selected important features between pectinolytic and non-pectinolytic genes in the training set. The *P*-values were calculated using the Wilcoxon test.

In addition to distinguishing pectinolytic genes from non-pectinolytic genes, ML also determines the relative importance of each feature when making a prediction. The most importance features for predicting pectinolytic genes in the RF model included co-expression with known pectinolytic genes, pI, the regulation by TFs AraR, GaaX and GaaR, and the gene expression profile during growth on pectin-related substrates (Fig. 3a). The importance ranking of these features are in line with the statistical comparison of their distribution between the positive and negative training set (Fig. 3b).

### Genome-wide prediction of pectinolytic genes

**Fig. 4.**
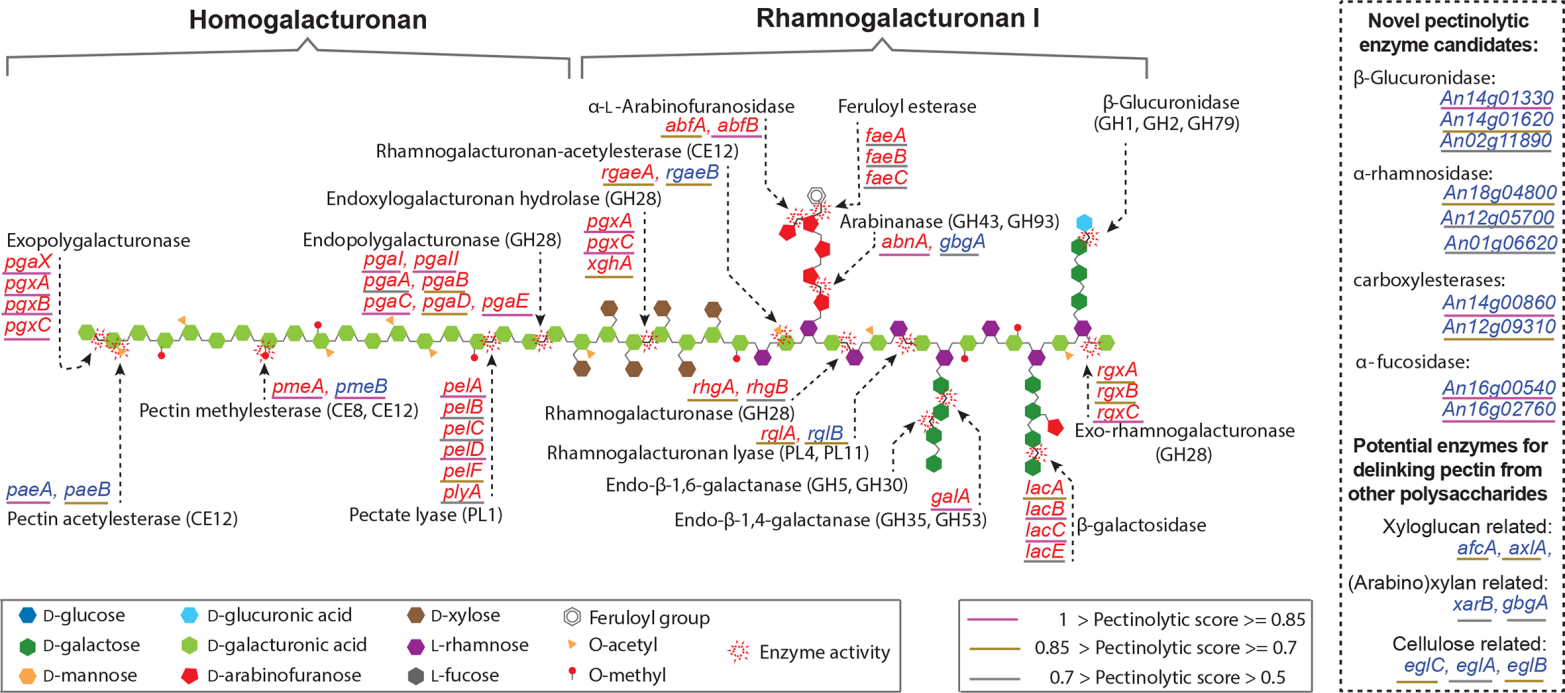
The inventory of *A. niger* genes encoding known and predicted pectinolytic enzymes with a schematic representation of their (predicted) activities on the pectin main and side chains. The names of characterized and potential pectinolytic genes identified in this study are indicated in red and blue type, respectively. The predicted pectinolytic score for each pectinolytic candidate obtained from the final Random Forest model is indicated with lines in different colour under the name of each gene. The purple, orange and grey line indicate high, medium and relative low pectinolytic scores, respectively. Novel enzyme candidates identified in this study are listed in the box at the right side of the figure. The schematic presentation of the polymers and enzyme activities was modified from the literature [56]. Note that this figure does not depict definitive molecular structures of pectin.

The RF model predicted 83 pectinase genes from the secretome of *A. niger*, consisting of 1533 genes with secretion signal (Table S6, Fig. 4). Based on the prediction outcomes, each gene was given a pectinolytic score ranging from 0 to 1 indicating the likelihood that the gene is a pectinolytic gene. All the 37 previously characterized pectinolytic genes and six of the nine previously predicted pectinolytic genes were successfully predicted from the secretome of *A. niger*. The 44 candidates with a relative high pectinolytic score (>0.7) mainly consist of 28 characterized genes, five previously predicted genes (*pmeB*, *rgaeB*, *rglB*, *paeA* and *paeB*), four genes encoding cellulose- and xyloglucan-degrading enzymes, and seven novel candidates. Two candidate genes (An14g00860 and An12g09310) contain a conserved carboxylesterase domain (PF00135), indicating that they may function as novel pectin-related methyl esterases, acetyl esterases or feruloyl esterases. Although there is no experimental evidence that the PF00135 domain is involved in plant polysaccharide degradation and the domain has not yet been linked to any known CAZy family, two previous studies have indicated their potential in plant biomass degradation [42, 43]. Two other candidates (An14g01330 and An14g01620) contain a GH79 domain, and are likely to function as a β-glucuronidase for removing d-glucuronic acid from the side chain of pectin [44]. Another novel candidate (An18g04800) belongs to GH78, and its homologue in *Penicillium chrysogenum* was previously reported as alpha-rhamnosidase (rhamnohydrolase) involved in pectin degradation [45]. The last two novel candidates (An16g00540 and An16g02760) belong to the CAZy GH95 family. GH95 represents the alpha-fucosidase enzyme that was able to attack rhamnogalacturonan II [46, 47], a minor quantitative component of pectin.

The 39 candidates with a relatively low pectinolytic score (<0.7) mainly consist of genes encoding plant polysaccharide-degrading enzymes and novel genes. In total, 17 genes encoding plant biomass-degrading (PBD) enzymes were identified in this list. In addition to nine characterized and one previously predicted pectinolytic genes, the other seven PBD genes were previously predicted as non-pectinolytic genes associated with decomposition of xyloglucan, (arabino-)xylan and cellulose. The enzymes encoded by these genes are unlikely to function as typical pectinolytic enzymes, but could play a role in de-linking pectin from other plant polysaccharides and allowing it to be more efficiently degraded. The novel genes consist of nine genes without any known predicted domain and 13 genes with conserved domains. It is of note that we identified two other GH78 genes (An12g05700 and An01g06620) and one GH79 gene (An02g11890). Another novel candidate (An11g02440) contains a galactose-binding domain in its sequence. Interestingly, this gene has not yet been included in the current CAZy database (http://www.cazy.org/), but its galactose-binding domain suggests it may assist in the release of galactose from pectin.

## Discussion

Compared to traditional enzyme discovery that mainly relies on searching for sequence homology and analysing a limited transcriptome data, the ML-based approach proposed in this study had several advantages. First, the ML model showed good performance in both recalling previously described pectinolytic enzymes and identifying novel candidate enzymes, while the conventional approach is restricted to identifying enzymes sharing considerable homology with the known enzymes. For instance, we identified two candidates (An14g00860 and An12g09310) encoding carboxylesterases, three candidates (An14g01330, An14g01620 and An02g11890) encoding glucuronidases, two candidates (An16g00540, An16g02760) encoding alpha-fucosidases, and three candidates (An18g04800, An12g05700 and An01g06620) encoding potential alpha-rhamnosidase. They do not belong to the same enzyme family as any of the previously known pectinolytic enzymes, but the predicted pectinolytic scores together with the conserved protein domains suggest strongly that they are good candidates for experimental validation. Compared to the results of a previous study using homology searches of known pectinolytic genes [9], our ML-based approach correctly excluded all 14 pseudo genes reported in the previous study. Among the other 46 previously predicted pectinolytic genes that were expressed, we successfully recalled 43 of them. The three miss-identified pectinolytic genes (*abnC*, *abnD* and *rhgC*) could be the result of false prediction in the previous study, or their different expression and regulation patterns compared to the known pectinolytic genes.

Second, we identified not only enzymes with a known or novel pectinolytic function, but also several groups of genes that have previously been described to encode enzymes involved in degrading non-pectin plant polysaccharides [e.g. xyloglucan, (arabino-)xylan and cellulose] (Table S6). The identification of these non-pectinolytic enzymes indicated that they were strongly co-expressed or co-regulated with the pectinolytic gens, which is in line with the synergistic interaction among different polysaccharides during fungal plant biomass degradation [48–50]. Given the complex covalent and non-covalent linkages between pectin and other polysaccharides present in plant cell walls [51–54], we hypothesize that the identified non-pectinolytic enzymes in this study could play a crucial role in de-linking pectin from other polysaccharides and/or exposing it to more efficient degradation. Although the degradation of plant polysaccharides is often discussed as an independent process, our results imply the importance of further investigation of the cooperative actions among different polysaccharide-degrading enzymes.

Among the six tested ML models, the RF performed better than the others. This is consistent with the performance of RF in other studies [15, 55]. In both the nested CV and independent test, RF correctly recognized over 94 % of negative instances, while the recall of positive instances was around 61 and 67 % in the nested CV and independent test, respectively. This indicates the challenge of new enzyme prediction based on a limit size of training set. Among the original described 46 pectinolytic genes, including 37 characterized pectinolytic genes, we successfully recalled all the 37 known genes and another six of the predicted pectinolytic genes. The candidate genes recalled in these two independent studies represent good targets for experimental validation.

Our study has demonstrated the tremendous potential of finding industrially important pectinolytic enzymes by integrative analysis of heterogeneous (post-)genomics features using the ML method. With the continuing expansion of (post-)genomics data on enzyme evolution, transcriptional expression and regulation, proteomics data, together with a better understanding of the physicochemical properties of enzymes, the ML-based bioinformatics approach will play an increasing role in mining big biological data, especially for the discovery of novel microbial enzymes.
